# Four Methods of Preparing mRNA 5′ End Libraries Using the Illumina Sequencing Platform

**DOI:** 10.1371/journal.pone.0101812

**Published:** 2014-07-08

**Authors:** Ryuji J. Machida, Ya-Ying Lin

**Affiliations:** Biodiversity Research Centre, Academia Sinica, Nankang, Taipei, Taiwan; VU University Medical Center, Netherlands

## Abstract

**Background:**

The 5′ untranslated regions of mRNA play an important role in their translation.

**Results:**

Here, we describe the development of four methods of profiling mRNA 5′ ends using the Illumina sequencing platform; the first method utilizes SMART (Switching Mechanism At 5′ end of RNA Transcript) technology, while the second involves replacing the 5′ cap structure with RNA oligomers via ligation. The third and fourth methods are modifications of SMART, and involve enriching mRNA molecules with (nuclear transcripts) and without (mitochondrial transcripts) 5′ end cap structures, respectively. Libraries prepared using SMART technology gave more reproducible results, but the ligation method was advantageous in that it only sequenced mRNAs with a cap structure at the 5′ end.

**Conclusions:**

These methods are suitable for global mapping of mRNA 5′ ends, both with and without cap structures, at a single molecule resolution. In addition, comparison of the present results obtained using different methods revealed the presence of abundant messenger RNAs without a cap structure.

## Introduction

Among the next-generation sequencing platforms, the Illumina platform offers by far the highest number of sequence reads per run. Illumina sequencing has been previously used for transcriptomic analyses e.g. [1], [2]. However, most available RNA-seq kits generate full length transcripts, rather than the 5′ end alone (e.g., NEBNext mRNA Library Prep (New England Biolabs); TruSeq (Illumina); ScriptSeq v2 RNA-Seq (epicentre) [3]). Furthermore, these kits require fragmentation of RNA templates before preparation of the sequencing library, which prohibits profiling of the 5′ end. Salimullah *et al.* recently described a 5′ end sequencing strategy called NanoCAGE [4]. This method is advantageous in that it requires only a small amount of total RNA as input. However, it uses random primers for the synthesis of first strand cDNA, which results in the amplification of non-adenylated RNA. A second strategy, called CAGE [5], also uses random primers for first strand synthesis [5], but its reliance on the EcoP15I restriction enzyme (which cleaves 27 bp away from the recognition site) limits the length of the resulting sequences to 27 nt. A third 5′ end sequencing strategy, called RAMPAGE, was more recently described by Batut *et al.* [6]. Advantages of RAMPAGE include the ability to identify capped transcription start sites and the potential for high sample number multiplexing; however, this method requires large quantities of total RNA (5 µg). Moreover, the Illumina platform requires the bases to be balanced at the beginning of each read (for cluster detection and cross-talk matrix generation during the first four cycles, and for phasing and pre-phasing rate calculations during cycles 2–12; [7]). None of the sequencing strategies described above meet this particular requirement.

In the present study, we developed four Illumina-based methods of preparing libraries for 5′ end profiling analysis. The first method is based on SMART (Switching Mechanism At 5′ end of RNA Transcript: [8]), while the second involves replacing the 5′ RNA cap structure with ligated RNA oligomers [9]. Libraries generated by the SMART method were found to be highly reproducible, allowing mRNA abundance to be measured directly based on sequence counts. In contrast, the ligation-based method enabled the mapping of 5′ end boundaries with mature cap structures. The resulting 5′ end profiles provide fresh insights into 5′ untranslated regions, revealing the presence of abundant mRNAs without a cap structure. The last two methods are modifications of SMART, used to enrich mRNA molecules with (CapSMART) or without cap (Non-CapSMART) structures. All four methods allow balanced representation of bases at the beginning of each read, which is required for high quality Illumina sequencing.

## Methods

Adult *Drosophila melanogaster* poly A+ RNA was purchased from a commercial source (Clontech: Cat. 636222, Lot. 1009305A). The cDNA libraries were constructed with the SMART cDNA Library Construction Kit (Clontech, Cat. 634901) (for the SMART method) or the ExactSTART Eukaryotic mRNA 5′- & 3′- RACE Kit (epicentre, Cat. ES80910) (for the ligation method), using a modified variant of the manufacturer's instructions (as described below). To determine reproducibility, six libraries were independently prepared using each SMART and ligation method, and multiplexed using single Illumina HiSeq lanes. Three libraries were also independently prepared using each CapSMART and Non-CapSMART method (each of the three libraries used different STOP oligos), and the resulting six libraries were multiplexed using a single Illumina lane. Following cDNA library construction, libraries generated by all four methods were subjected to the same workflow, which involved sonication, biotin collection of the 5′ end, and Illumina library preparation. All thermal reactions were performed using a Veriti thermal cycler (Applied Biosystems).

To further confirm the reproducibility of each method, a single SMART library and four ligation libraries (using tags TAG02, TAG04, TAG05, and TAG06) were also constructed using embryonic *Drosophila melanogaster* poly A+ RNA (Clontech: Cat. 636224, Lot. 1210373A). The four ligation libraries were pooled before sequencing. Illumina MiSeq was used to sequence the pooled ligation libraries and the SMART library. With the exception of the sequencing machine, all experiments were performed as described for the adult poly A+ samples (above). Sequence out put from all four ligation libraries were pooled before analyses. The first 101 nucleotides of the sequence output were used for further genome mapping analyses.

### Preparation of cDNA libraries using the SMART method

**Figure 1:**
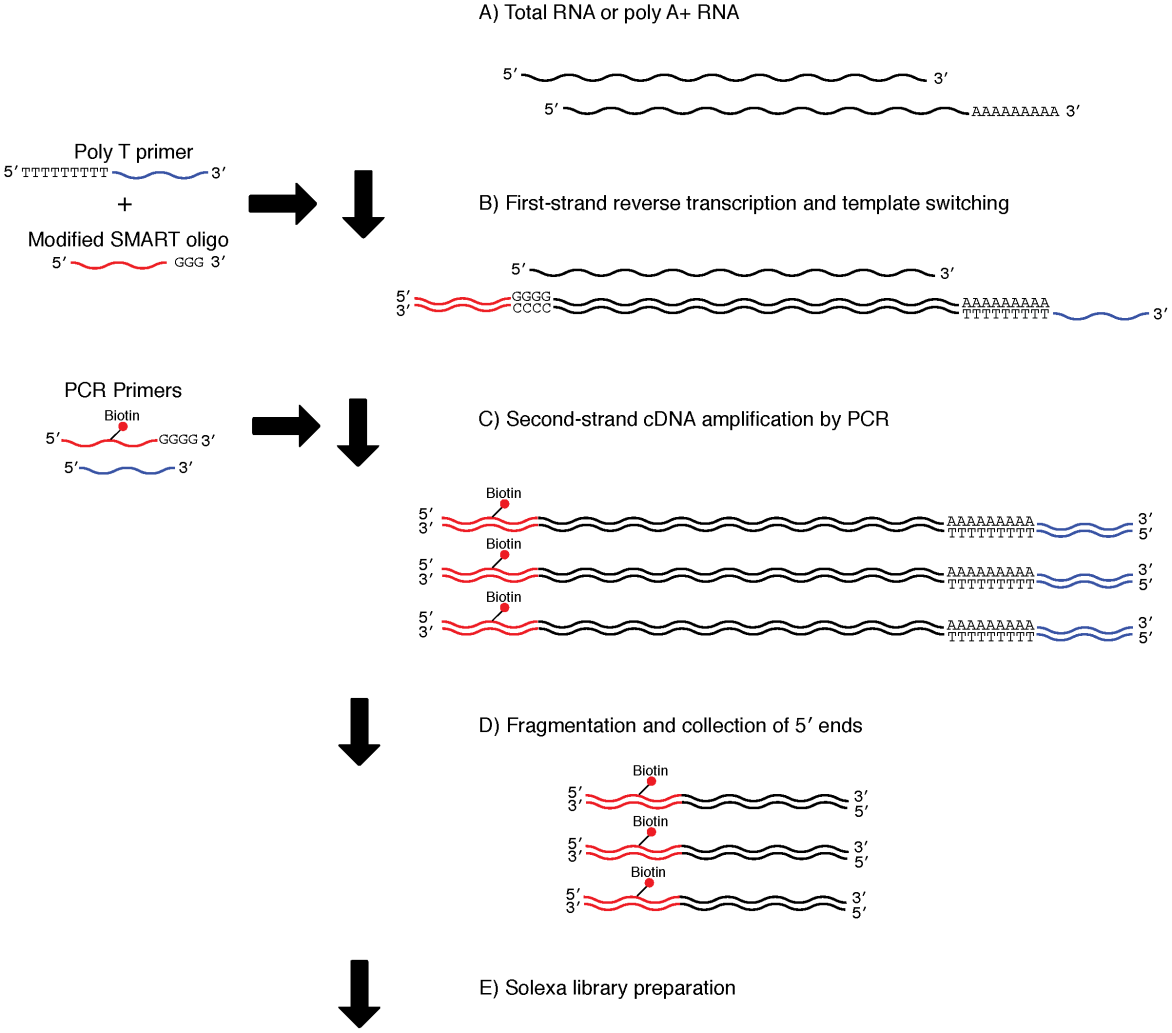
Library preparation using the SMART method. A) The protocol used either poly A+ (0.025–0.5 µg) or total (0.05–1.0 µg) RNA. B) First-strand cDNA synthesis, together with template switching and continuous replication to the end of the oligonucleotide. C) Second-strand cDNA amplification by PCR with biotinylated 5′ end primers. D) Fragmentation of cDNA using a Bioruptor and collection of biotinylated 5′ ends using beads. E) Illumina sequencing library preparation.

Libraries were constructed using the SMART cDNA Library Construction Kit (Clontech) with modified SMART oligonucleotides (Table 1, Fig. 1). First, a 5 µl reaction volume containing 375 ng of poly A+ RNA (Clontech), 1 µl of modified SMART oligonucleotide {Table 1, Modified SMART (12 µM): AGA GTG TTT GGG TAG AGC AGC GTG TTG GCA TGT ggg (lower case for RNA), synthesized and HPLC purified by Metabion, Germany} and 1 µl of CDS III/3′ PCR primer was incubated for 2 minutes at 72°C to denature the RNA. The tube was placed on ice for 2 minutes immediately after the incubation. First-strand reverse transcription was subsequently performed by adding 2 µl of 5× First-strand buffer, 1 µl of DTT, 1 µl of dNTP (2.5 mM each), and 1 µl of SMARTScribe MMLV Reverse Transcriptase (with the exception of the modified SMART oligo, all chemicals are included in Clontech SMART kit) to the tube, and incubating it for 60 minutes at 42°C.

**Table 1:** List of modified oligonucleotides used for SMART, CapSMART and Non-CapSMART.

Steps in experiment workflow	Oligonucleotide name	Sequence^a^
First-strand reverse transcription	Modified SMART	AGAGTGTTTGGGTAGAGCAGCGTGTTGGCATGTggg
Second-strand cDNA amplification	Modified CDS III/3′	ATTCTAGAGGCCGAGGCGGCCGACATG
Second-strand cDNA amplification	SMART 5′ biotin	AGAGTGTTTGGGTAGAGCAGCG_T_GTTGGCATGTGGG * G
Ligation of STOP Oligo	STOP1	iGiCiG
Ligation of STOP Oligo	STOP2	iCiGiC

All oligonucleotides were purified by HPLC, except for STOP oligos.

a 5′ - 3′; lower-case letters indicate RNA oligonucleotides; subscript indicates biotinylation;

* indicates a PTO bond; i indicates isomers.

After reverse transcription, the resulting cDNA was amplified by LD PCR (Clontech). Each PCR consisted of a 50 µl reaction volume containing 37 µl of nuclease-free water, 5 µl of 2 PCR buffer, 4 µl of dNTP (2.5 mM each), 1 µl of modified CDS III/3′ primer (Table 1, Modified CDS III/3′ (12 µM): ATT CTA GAG GCC GAG GCG GCC GAC ATG, synthesized and HPLC purified by Genomics BioSci & Tech, Taiwan), 1 µl of biotinylated primer {Table 1, SMART 5′ biotin (12 µM): AGA GTG TTT GGG TAG AGC AGC G_T_G TTG GCA TGT GGG *G (subscript indicates biotinylation, * indicates a PTO bond), synthesized and HPLC purified by SynGen, USA}, 1 µl of Advantage 2 DNA Polymerase Mix, and 1 µl of the first-strand reverse transcript product (with the exception of the modified CDS III/3′ primer, biotinylated primer, and dNTP, all chemicals are included in the Clontech SMART kit). Initial denaturation was carried out at 95°C for 10 min, followed by 22 cycles of the following thermal-cycle profile: denaturation at 95°C for 5 seconds, annealing and extension at 68°C for 6 minutes. The resulting products were electrophoresed on a 1% TAE agarose gel together with Safe-Green (Applied Biological Materials Inc.), and visualized using a blue light transilluminator (Maestrogen: LB-16). PCR products were purified by Agencourt AMPure XP (Beckman Coulter) and eluted in 150 µl of Elution Buffer (Qiagen).

### Preparation of cDNA libraries using the ligation method

**Figure 2:**
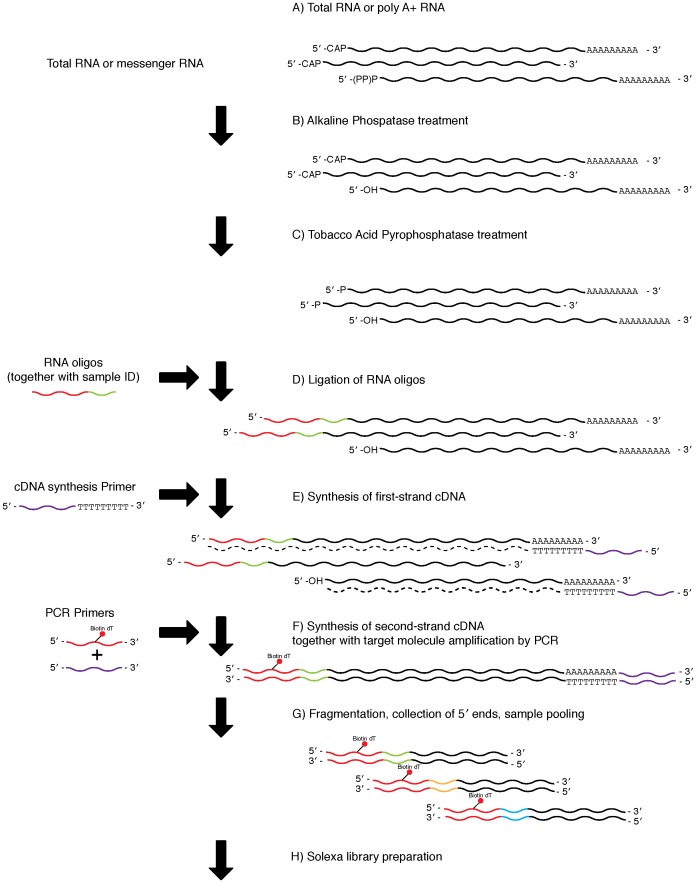
Library preparation using the ligation method. A) The protocol used either poly A+ (0.50–10 µg) or total (10–200 µg) RNA. B) De-phosphorylation of mono-, di-, and tri- phosphate groups from non-capped 5′ end molecules using alkaline phosphatase. C) Tobacco Acid Pyrophosphatase treatment to remove the 5′ cap structure, exposing a mono-phosphate group for subsequent ligation. D) Ligation of RNA oligomers. A total of six tags (Table 3: TAG02, TAG04, TAG05, TAG06, TAG07, TAG12) were used in the present study. E) First-strand cDNA synthesis. F) Second-strand cDNA amplification by PCR with biotinylated 5′ end primers. G) Fragmentation of cDNA using a Bioruptor, collection of biotinylated 5′ ends using beads, and sample pooling for multiplexing. H) Illumina sequencing library preparation.

Libraries were constructed using the ExactSTART Eukyaryotic mRNA 5′- & 3′- RACE Kit (epicentre) with modified 5′-RACE Acceptor Oligos (Table 2, Fig. 2). First, alkaline phosphatase was used to remove the 5′-phosphate group from 5′- mono-, di-, and tri-phosphorylated RNAs: a 100 µl reaction volume containing 375 ng of poly A+ RNA, 10 µl of APex Reaction Buffer, 5 µl of APex Heat-Labile Alkaline Phosphatase, and nuclease-free water was incubated for 15 minutes at 37°C. After the reaction, the products were purified using the RNeasy MinElute Cleanup kit (Qiagen), and eluted with 10 µl nuclease-free water. The products were then treated with Tobacco Acid Pyrophosphatase (TAP) to remove the 5′ cap structure and expose a mono-phosphate group for ligation; a reaction mixture consisting of 1 µl of TAP buffer, 0.5 µl of RiboGuard RNase Inhibitor, 1 µl of TAP enzyme, and 7.5 µl of alkaline phosphatase-treated RNA was incubated for 30 minutes at 37°C. Next, 10 µl of TAP-treated RNA were incubated with 4 µl of nuclease-free water, 2 µl of RNA ligase buffer, 1 µl of TAP STOP buffer, 1 µl of modified 50 µM 5′-RACE Acceptor Oligo, 1 µl of 2 mM ATP solution, and 1 µl of T4 RNA ligase for 30 minutes at 37°C to ligate modified 5′-RACE Acceptor Oligos to the RNA. This step required thorough mixing of the reaction after the addition of STOP buffer and before the addition of ATP solution. Each reaction contained one of six different modified 5′ -RACE Acceptor Oligos (Table 2: TAG02, TAG04, TAG05, TAG06, TAG07, TAG12). It is important to select appropriate sets of oligomers for high sequence quality [10]. Following ligation, first-strand reverse transcription was performed by adding 14 µl of nuclease-free water, 1 µl of cDNA synthesis primer, 2 µl of dNTP PreMix (2.5 mM each), 2 µl of MMLV RT buffer, and 1 µl of MMLV Reverse Transcriptase to the RNA, and incubating the reaction for 60 minutes at 37°C, followed by 10 minutes at 85°C. RNase digestion was then performed by adding 1 µl of RNase solution to the reaction mixture for 5 minutes at 55°C (all chemicals used are included in the epicentre ExactSTART Eukaryotic mRNA 5′-&3′- RACE Kit, with the exception of the modified 5′-RACE Acceptor Oligos).

**Table 2:** List of modified oligonucleotides used for the ligation method.

Steps in experiment workflow	Oligonucleotide name	Sequence^a^
5′-RACE Acceptor Oligo ligation	TAG01	agaguguuuggguagagcagcguguuggcaugu*aucacg*
	TAG02	agaguguuuggguagagcagcguguuggcaugu*cgaugu*
	TAG03	agaguguuuggguagagcagcguguuggcaugu*uuaggc*
	TAG04	agaguguuuggguagagcagcguguuggcaugu*ugacca*
	TAG05	agaguguuuggguagagcagcguguuggcaugu*acagug*
	TAG06	agaguguuuggguagagcagcguguuggcaugu*gccaau*
	TAG07	agaguguuuggguagagcagcguguuggcaugu*cagauc*
	TAG08	agaguguuuggguagagcagcguguuggcaugu*acuuga*
	TAG09	agaguguuuggguagagcagcguguuggcaugu*gaucag*
	TAG10	agaguguuuggguagagcagcguguuggcaugu*uagcuu*
	TAG11	agaguguuuggguagagcagcguguuggcaugu*ggcuac*
	TAG12	agaguguuuggguagagcagcguguuggcaugu*cuugua*
Second-strand cDNA amplification	Ligation 5′ biotin	AGAGTGTTTGGGTAGAGCAGCG_T_GTTGGCATGT

All oligonucleotides were purified by HPLC.

a 5′ - 3′; lower-case letters indicate RNA oligonucleotides; italicized sequences are unique for each tag; subscript indicates biotinylation.

After RNase digestion, second-strand cDNA synthesis was performed by PCR, by setting up a 50 µl reaction volume containing 13 µl nuclease-free water, 5 µl of 2 PCR buffer (Clontech), 4 µl of dNTP (2.5 mM each), 2.5 µl of PCR primer 2 (epicentre, ExactSTART kit), 2.5 µl of biotinylated primer {Table 2, Ligation 5′ biotin (2 µM): AGA GTG TTT GGG TAG AGC AGC G_T_G TTG GCA TGT (subscript indicates biotinylation), synthesized and HPLC purified by SynGen, USA}, 2.5 µl of Advantage 2 DNA Polymerase Mix (Clontech), and 20.5 µl of first-strand reverse transcript product. PCR amplification was confirmed by electrophoresis, as described in the previous section. After purification using Agencourt AMPure XP, samples were eluted with 30 µl of Elution Buffer, and the dsDNA concentration was measured using the Qubit dsDNA HS Assay Kit (Invitrogen). Equal amounts of quantified libraries were pooled in a single tube, and the volume was adjusted to 150 µl with nuclease-free water.

### Preparation of cDNA libraries using the CapSMART method

**Figure 3:**
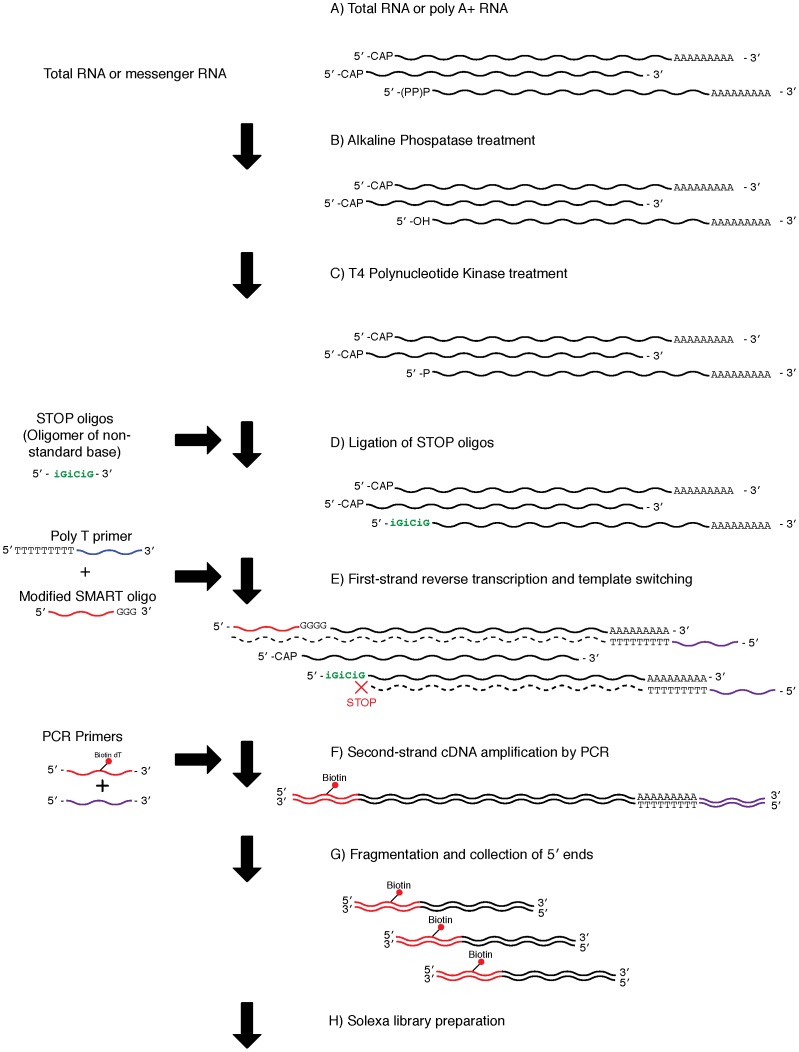
Library preparation using the CapSMART method. A) The protocol used either poly A+ (0.50–10 µg) or total (10–200 µg) RNA. B) De-phosphorylation of mono-, di-, and tri- phosphate groups from non-capped 5′ end molecules using alkaline phosphatase. C) Phosphorylation to add mono-phosphate to the non-capped 5′ end molecules using T4 Polynucleotide Kinase. D) Ligation of STOP oligos. A total of three kinds of oligonucleotides (Table 2: STOP1: iGiCiG, STOP2: iCiGiC, STOPMix: mixture of STOP1 and STOP2) were used in the present study. E) First-strand cDNA synthesis. F) Second-strand cDNA amplification by PCR with biotinylated 5′ end primers. G) Fragmentation of cDNA using a Bioruptor and collection of biotinylated 5′ ends using beads. H) Illumina sequencing library preparation.

Libraries were constructed using both ExactSTART Eukyaryotic mRNA 5′- & 3′- RACE (epicentre) and SMART cDNA library Construction Kits (Clontech), with modified SMART oligonucleotides and STOP oligos (Table 1, Fig. 3). First, alkaline phosphatase was used to remove the 5′-phosphate group from 5′- mono-, di-, and tri-phosphorylated RNAs: a 100 µl reaction volume containing 375 ng of poly A+ RNA, 10 µl of APex Reaction Buffer, 5 µl of APex Heat-Labile Alkaline Phosphatase, and nuclease-free water was incubated for 15 minutes at 37°C. After the reaction, the products were purified using the RNeasy MinElute Cleanup kit (Qiagen), and eluted with 18 µl nuclease-free water. The products were then treated with T4 Polynucleotide Kinase to add mono-phosphate to non-capped mRNA to ready it for ligation; a reaction mixture consisting of 1 µl of T4 Polynucleotide Kinase (Fermentas, # EK0032), 2 µl of RNA Ligase Reaction Buffer (New England Biolabs), 0.5 µl of RNaseOUT (Invitrogen, #10777-019), 1 µl of 100 mM ATP solution (Fermentas, #R0441), and 15.5 µl of alkaline phosphatase-treated RNA was incubated for 30 minutes at 37°C. Next, 20 µl of T4 Polynucleotide Kinase-treated RNA were incubated with 2.5 µl of nuclease-free water, 1 µl of RNA Ligase Reaction Buffer (New England Biolabs), 4.5 µl of PEG8000 (New England Biolabs), 1 µl of STOP oligo {Table 1, STOP1 (50 µM): iGiCiG, STOP2 (50 µM): iCiGiC, STOP Mix (50 µM): mixture of STOP1 and STOP2, synthesized by Metabion, Germany}, and 1 µl of T4 RNA Ligase (New England Biolabs, M0204S) for 16 hours at 16°C to ligate STOP oligos to the non-capped mRNA. To test ligation bias of the STOP oligos, three reactions were performed, using STOP1, STOP2, and STOP Mix. Following STOP oligo ligation, the products were purified using the RNeasy MinElute Cleanup Kit (Qigaen), and eluted with 10 µl nuclease-free water. Subsequently, 3 µl of these purified products were incubated together with 1 µl of modified SMART oligonucleotide {Table 1, Modified SMART (12 µM)} and 1 µl of CDS III/3′ PCR primer for 2 minutes at 72°C to denature the RNA. The tube was placed on ice for 2 minutes immediately after incubation. First-strand reverse transcription was subsequently performed by adding 2 µl of 5× First-strand buffer, 1 µl of DTT, 1 µl of dNTP (2.5 mM each), and 1 µl of SMARTScribe MMLV Reverse Transcriptase to the tube, and incubating it for 60 minutes at 42°C. After reverse transcription, the resulting cDNA was amplified by LD PCR (Clontech), as described above.

### Preparation of cDNA libraries using the Non-CapSMART method

**Figure 4:**
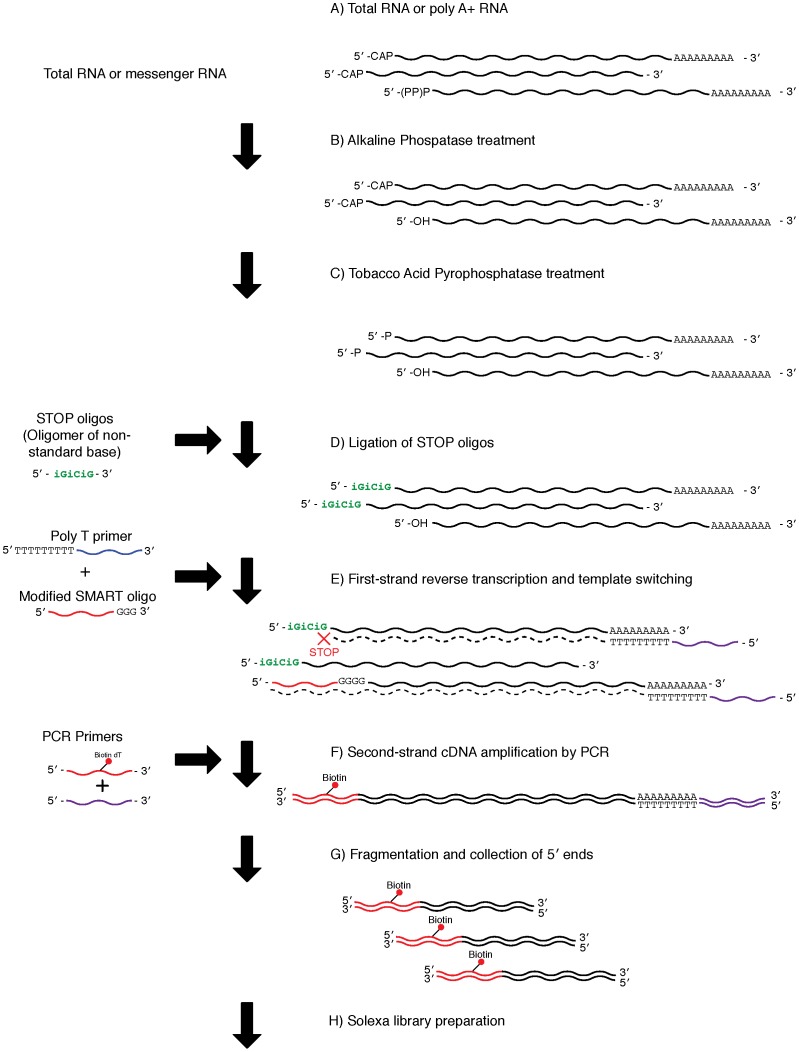
Library preparation using the Non-CapSMART method. A) The protocol used either poly A+ (0.50–10 µg) or total (10–200 µg) RNA. B) De-phosphorylation of mono-, di-, and tri- phosphate groups from non-capped 5′ end molecules using alkaline phosphatase. C) Tobacco Acid Pyrophosphatase treatment to remove the 5′ cap structure, exposing a mono-phosphate group for subsequent ligation. D) Ligation of STOP oligos. A total of three kinds of oligonucleotides (Table 2: STOP1: iGiCiG, STOP2: iCiGiC, STOPMix: mixture of STOP1 and STOP2) were used in the present study. E) First-strand cDNA synthesis. F) Second-strand cDNA amplification by PCR with biotinylated 5′ end primers. G) Fragmentation of cDNA using a Bioruptor and collection of biotinylated 5′ ends using beads. H) Illumina sequencing library preparation.

Libraries were constructed using both the ExactSTART Eukyaryotic mRNA 5′- & 3′- RACE (epicentre) and SMART cDNA library Construction Kits (Clontech), with modified SMART oligonucleotides and STOP oligos (Table 1, Fig. 4). First, alkaline phosphatase was used to remove the 5′-phosphate group from 5′- mono-, di-, and tri-phosphorylated RNAs: a 100 µl reaction volume containing 375 ng of poly A+ RNA, 10 µl of APex Reaction Buffer, 5 µl of APex Heat-Labile Alkaline Phosphatase, and nuclease-free water was incubated for 15 minutes at 37°C. After the reaction, the products were purified using the RNeasy MinElute Cleanup kit (Qiagen), and eluted with 10 µl nuclease-free water. The products were then treated with Tobacco Acid Pyrophosphatase (TAP) to remove the 5′ cap structure and expose a mono-phosphate group for ligation; a reaction mixture consisting of 1 µl of TAP buffer, 0.5 µl of RiboGuard RNase Inhibitor, 1 µl of TAP enzyme, and 7.5 µl of alkaline phosphatase-treated RNA was incubated for 30 minutes at 37°C. Next, 10 µl of TAP-treated RNA were incubated with 4 µl of nuclease-free water, 2 µl of RNA ligase buffer, 1 µl of TAP STOP buffer, 1 µl of STOP Oligo (Table 1), 1 µl of 2 mM ATP solution, and 1 µl of T4 RNA ligase for 16 hours at 16°C to ligate modified STOP Oligos to the RNA. This step required thorough mixing of the reaction after the addition of STOP buffer and before the addition of ATP solution. To test ligation bias of the STOP oligos, three reactions were performed, using STOP1, STOP2, and STOP Mix. Following the STOP oligo ligation, the products were purified using the RNeasy MinElute Cleanup Kit (Qigaen), and eluted with 10 µl nuclease-free water. Next, 3 µl of the purified products were incubated together with 1 µl of modified SMART oligonucleotide {Table 1, Modified SMART (12 µM)} and 1 µl of CDS III/3′ PCR primer for 2 minutes at 72°C to denature the RNA. The tube was placed on ice for 2 minutes immediately after the incubation. First-strand reverse transcription was subsequently performed by adding 2 µl of 5× First-strand buffer, 1 µl of DTT, 1 µl of dNTP (2.5 mM each), and 1 µl of SMARTScribe MMLV Reverse Transcriptase to the tube, and incubating it for 60 minutes at 42°C. After reverse transcription, the resulting cDNA was amplified by LD PCR (Clontech), as described above.

### Sonication and biotin collection of 5′ ends

The following procedures were carried out for all libraries. Sonication was performed using a Bioruptor (Diagenode). Prepared libraries were sonicated by 20 cycles of ON/OFF for 30 seconds each at high intensity. Sonicated products were electrophoresed to confirm fragmentation.

Biotinylated 5′ ends were subsequently collected using 50 µl of Dynabeads MyOne Streptavidin T1 (Invitrogen) per reaction, following the manufacturer's protocol. Each reaction consisted of either 50 µl of purified SMART, CapSMART, and Non-CapSMART method-generated products or 100 µl of purified ligation method-generated products. In brief, samples were immobilized and washed, and then incubated with 50 µl elution buffer (1 µl 0.5 M EDTA, 47.5 µl formamide, 1.5 µl H_2_O) for 5 minutes at 65°C. Further purification was performed using a 90% volume (45 µl) of Agencourt AMPure XP, and 5′ ends were eluted with 30 µl Elution Buffer for SMART, CapSMART, and Non-CapSMART products, or 60 µl for ligation method products.

### Library preparation and Illumina sequencing

Illumina sequencing libraries were prepared following Meyer & Kircher [10]. In brief, biotin-collected 5′ ends were subjected to blunt-end repair (100 ng of collected 5′ ends were used for each preparation). Next, Illumina-compatible adaptors were ligated to the 5′ ends, and adaptor-gaps were filled in. After each reaction, products were purified using Agencourt AMPure XP. After the final purification, indexing PCR was performed by adding 26.5 µl of nuclease-free water, 4 µl of dNTP (25 mM each), 5 µl of 2 PCR buffer, 1 µl of IS4_5′ primer (10 µM) {modified from IS4, Table 3: AAT GAT ACG GCG ACC ACC GAG ATC TAC ACT CTT TCC CTA CAC GAC GCT CTT CCG ATC TAG AGT GTT TGG G*T (* indicates a PTO bond), synthesized and HPLC purified by Metabion, Germany}, 1 µl of indexing primer (10 µM), 2.5 µl of Advantage 2 DNA Polymerase Mix (Clontech), and 10 µl of Illumina adaptor-ligated product to a total reaction volume of 50 µl. Modified IS4 primer was used to enable directional sequencing. For SMART, CapSMART, and Non-CapSMART library samples, indexing PCR was performed using six different indexing primers for multiplexing (indexing primers: ID01, ID02, ID03, ID04, ID05, and ID06 [10]). Six SMART libraries were independently indexed and pooled as a single sample. Three CapSMART and three Non-CapSMART libraries were also independently indexed and pooled as a single sample. For indexing PCR, initial denaturation was carried out at 95°C for 10 min, followed by 12 cycles of the following thermal-cycle profile: denaturation at 95°C for 20 seconds, annealing at 60°C for 20 seconds, and extension at 72°C for 30 seconds. PCR amplification was confirmed by electrophoresis, as described previously. PCR products were purified by Agencourt AMPure XP and eluted using 30 µl of Elution Buffer (Qiagen). Libraries independently prepared using SMART, CapSMART and Non-CapSMART technology were quantified using the Qubit dsDNA HS Assay Kit. Equal amounts of SMART quantified libraries were pooled in a single tube, and sent for sequencing (Sequencing Core, Biodiversity Research Centre, Academia Sinica). In addition, libraries equivalent to 300 µg of CapSMART and to 50 µg of Non-CapSMART were pooled separately in single tubes, and sent for sequencing.

**Table 3:** List of modified oligonucleotides used for Illumina sequencing library preparation, and custom sequencing primers for the SMART and ligation methods.

Steps in experiment workflow	Oligonucleotide name	Sequence^a^
Indexing PCR	IS4_5′	AATGATACGGCGACCACCGAGATCTACACTCTTTCCCTACACGACGCTCTTCCGATCTAGAGTGTTTGGG * T
Sequencing	Custom sequencing primer for SMART, CapSMART, and Non-CapSMART method	TGTTTGGGTAGAGCAGCGTGTTGGCATGTGGGG
Sequencing	Custom sequencing primer for ligation method	AGAGTGTTTGGGTAGAGCAGCGTGTTGGCATGT

All oligonucleotides were purified by HPLC.

a 5′ - 3′;

* indicates a PTO bond.

Sequencing was performed using the following Illumina instruments: HiSeq for adult poly A+ and MiSeq for embryo poly A+ samples, in accordance with the manufacturer's protocol (with the exception of the use of a modified read 1 sequencing primer). Different custom sequencing primers were used depending on the library preparation method (Table 3: custom sequencing primers for SMART, CapSMART and Non-CapSMART method: TGT TTG GGT AGA GCA GCG TGT TGG CAT GTG GGG; custom sequencing primer for ligation method: AGA GTG TTT GGG TAG AGC AGC GTG TTG GCA TGT, synthesized and HPLC purified by Genomics BioSci & Tech, Taiwan).

### Sequence analysis

Reproducibility tests were performed using Fastx-Toolkit, MySQL, and Perl scripts, as described below. First, sequences obtained from pooled samples using the ligation method were separated into different sample sources based on unique TAG sequences (Table 2: TAG02, TAG04, TAG05, TAG06, TAG07, TAG12) using fastx_barcode_splitter.pl, and allowing a single mismatch. Next, frequencies of identical sequences from each library were counted using fastx_collapser. After tidying the data format using Perl script, datasets were imported into MySQL, and the frequencies of identical sequences between libraries were extracted. In this comparison, sequences which occurred less than 10 times were disregarded. This reproducibility test was performed for adult poly A+ RNA libraries prepared by both SMART and ligation methods.

Mapping and data filtration of reads were performed using Bowtie2 [11] and MySQL, as follows. First, reads from each library were mapped onto the *Drosophila melanogaster* genome (Release 5: [12]) using Bowtie2 with the default settings. Next, output SAM files were imported into MySQL, and positional information (counts for each position) was extracted using MySQL commands. Only those sequences that mapped onto the minus strand of chromosome 2L were analyzed in the present study.

Mitochondrial transcript frequency was estimated for adult poly A+ RNA libraries as follows. First, fastx_collapser command (Fastax-tool kit) was performed for each library. Next, the output files were imported into local BLAST+ [13] and used as reference sequence. BLAST searches were subsequently performed using the first 100 bp of each mitochondrial gene sequence as query. BLAST was performed using the default settings, but the dust option was disabled, and seeding word size was set to 50 bp.

## Results and Discussion

Six adult poly A+ RNA libraries were prepared using both the SMART and ligation methods, and each of the six libraries were pooled and analyzed in two lanes. The CapSMART and Non-CapSMART methods were used to prepare three libraries each, and the six resulting libraries were pooled and analyzed in a single lane. Using three lanes of an Illumina HiSeq sequencer, the SMART method generated a total of 164,160,619 reads, the ligation method generated a total of 130,314,839 reads, and the CapSMART and Non-CapSMART methods generated a total of 150,847,202 reads (Table 4). Embryonic poly A+ RNA was used to prepare one library using the SMART method, and four libraries using the ligation method. Using an Illumina MiSeq sequencer, the SMART method generated 8,461,669 reads and the ligation method generated a total of 9,688,990 reads.

**Table 4:** Read numbers of sequences obtained from multiplexed samples using adult poly A+ RNA.

SMART						
Total	ID01	ID02	ID03	ID04	ID05	ID06
164,160,619	25,882,115	26,062,243	28,514,523	28,076,062	27,965,537	27,660,139

Ligation						
Total	TG02	TG04	TG05	TG06	TG07	TG12
130,314,839	28,913,869	22,271,629	26,246,399	14,913,150	19,706,734	18,263,058

	CapSMART			NonCapSMART		
Total	ID01	ID02	ID03	ID04	ID05	ID06
150,847,202	33,861,079	44,819,643	43,918,666	9,652,581	9,492,795	9,102,438

### Reproducibility

**Figure 5:**
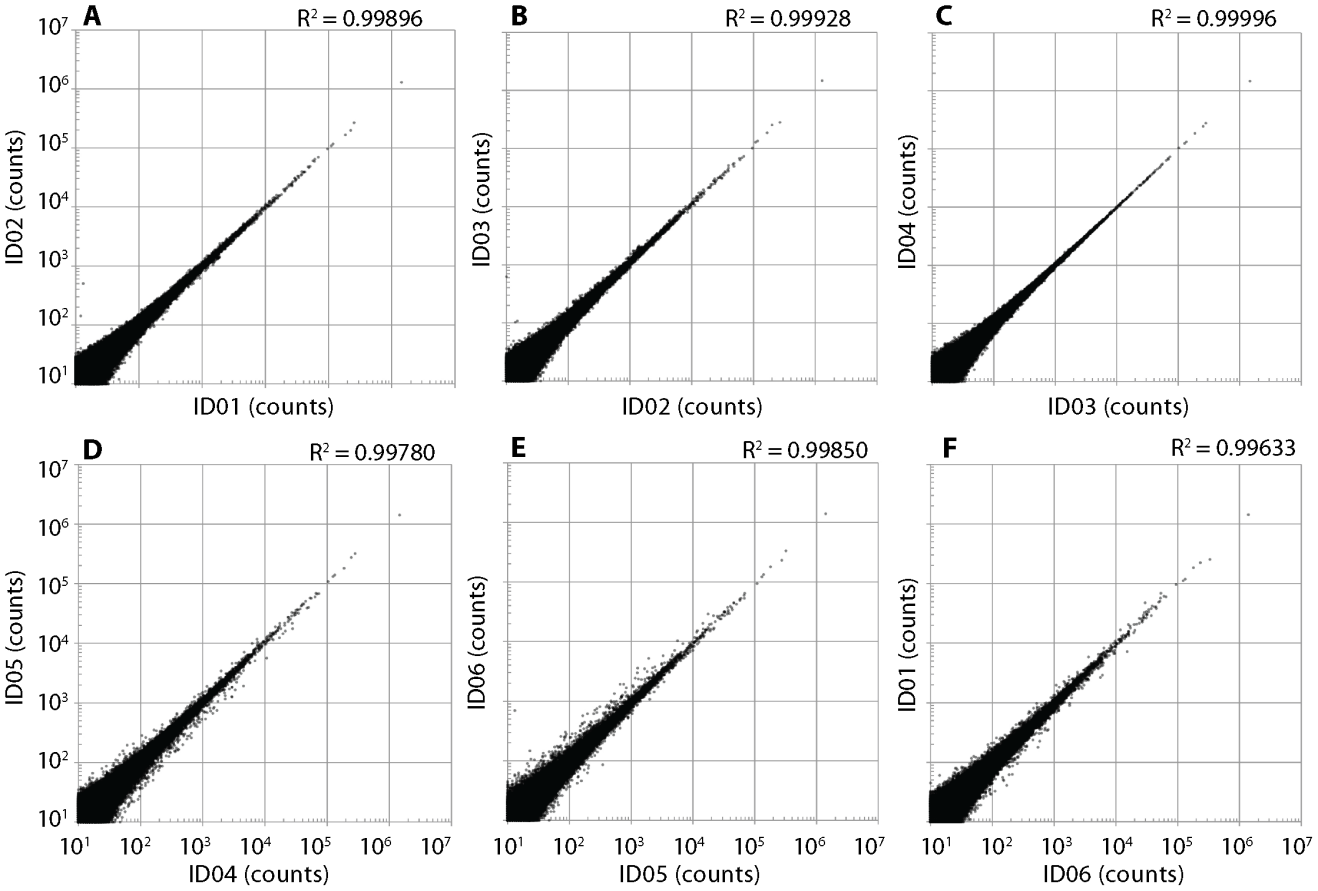
Reproducibility of libraries generated by the SMART method using adult poly A+ RNA. Plots showing correlation between sequence counts of six independent replicates (A: ID01 X 02; B: ID02 X 03; C: ID03 X 04; D: ID04 X 05; E: ID05 X 06; F: ID06 X 01). The results show a very high correlation (*R* = 0.99633–0.99996), indicating that the library preparation method is highly reproducible.

**Figure 6:**
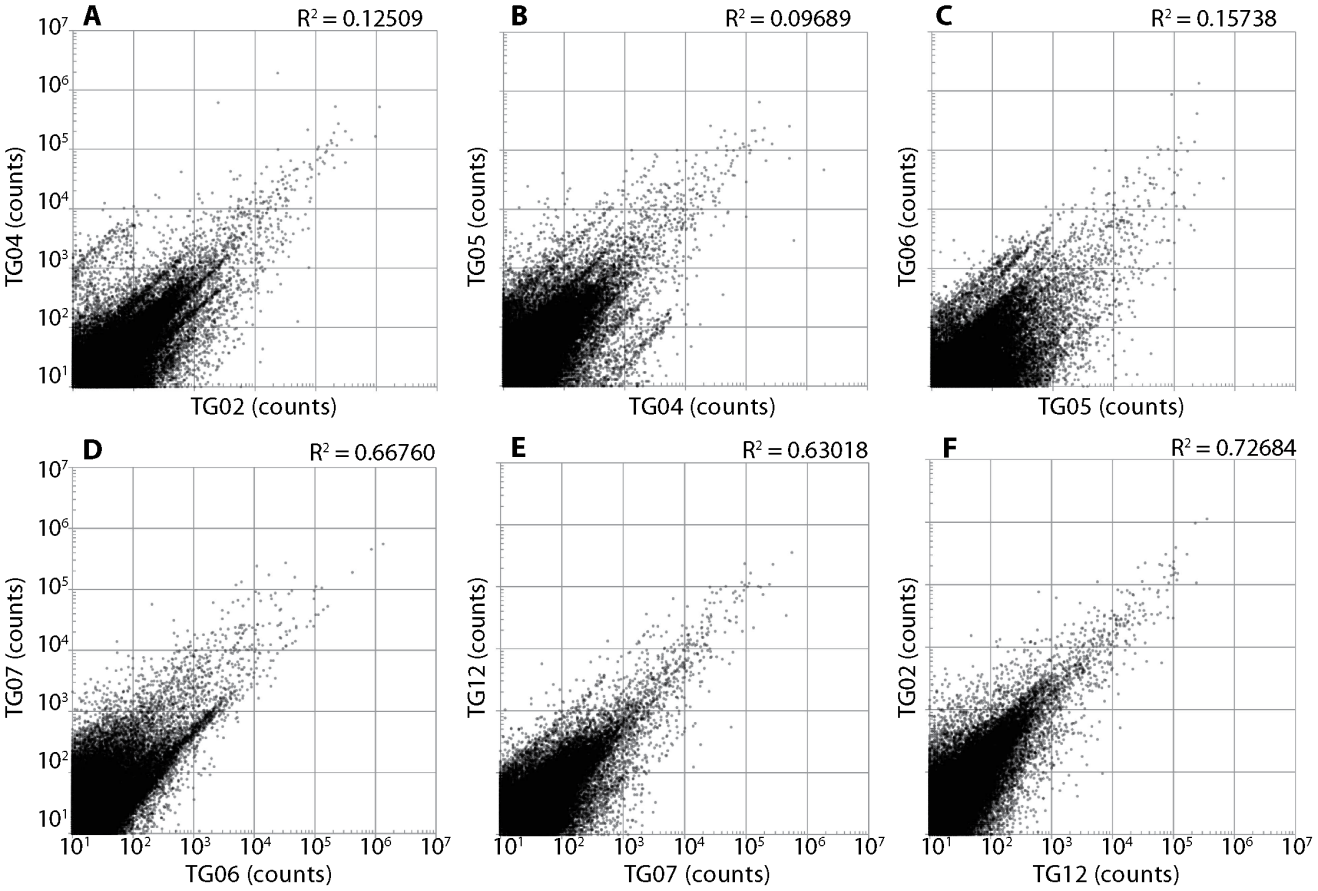
Reproducibility of libraries generated by the ligation method using adult poly A+ RNA. Plots showing correlation between sequence counts of six independent replicates (A: TG02 X 04; B: TG04 X 05; C: TG05 X 06; D: TG06 X 07; E: TG07 X 12; F: TG12 X 02). The results show a relatively low correlation (*R* = 0.09689–0.72684), indicating low reproducibility of the library preparation method.

Reproducibility was determined by comparing the frequencies of the same sequences between SMART- and ligation-derived libraries (Fig. 5 and 6). High reproducibility was observed for libraries prepared by the SMART method (Fig. 5). In contrast, relatively poor reproducibility was observed for libraries prepared by the ligation method (Fig. 6). The large amount of bias observed using the ligation method is assumed to be caused by ligation bias [14], [15], [16].

### Distribution of 5′ ends

**Figure 7:**
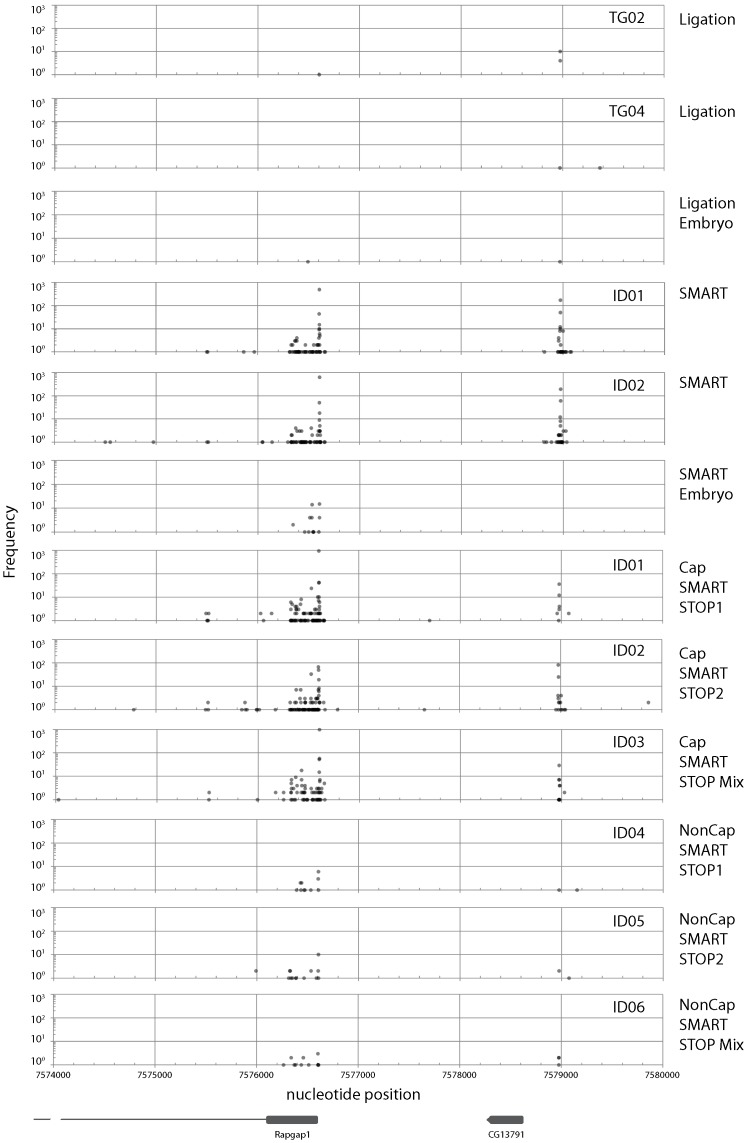
Frequency distribution of mapped sequence reads on the *Drosophila melanogaster* genome (Release 5) between nucleotide positions 7574000 and 7580000 on chromosome 2L. Only the sequences mapped on the minus strand are depicted. Gene locations (*Rapgap1* and *CG13791*) are depicted at the bottom of the figure. Plots from three libraries using SMART and ligation methods {adult (TG02, TG04, ID01, and ID02) and embryo RNA} and from three libraries using CapSMART and Non-CapSMART methods (ID01, ID02, ID03, ID04, ID05, and ID06) are depicted in the figure.

**Figure 8:**
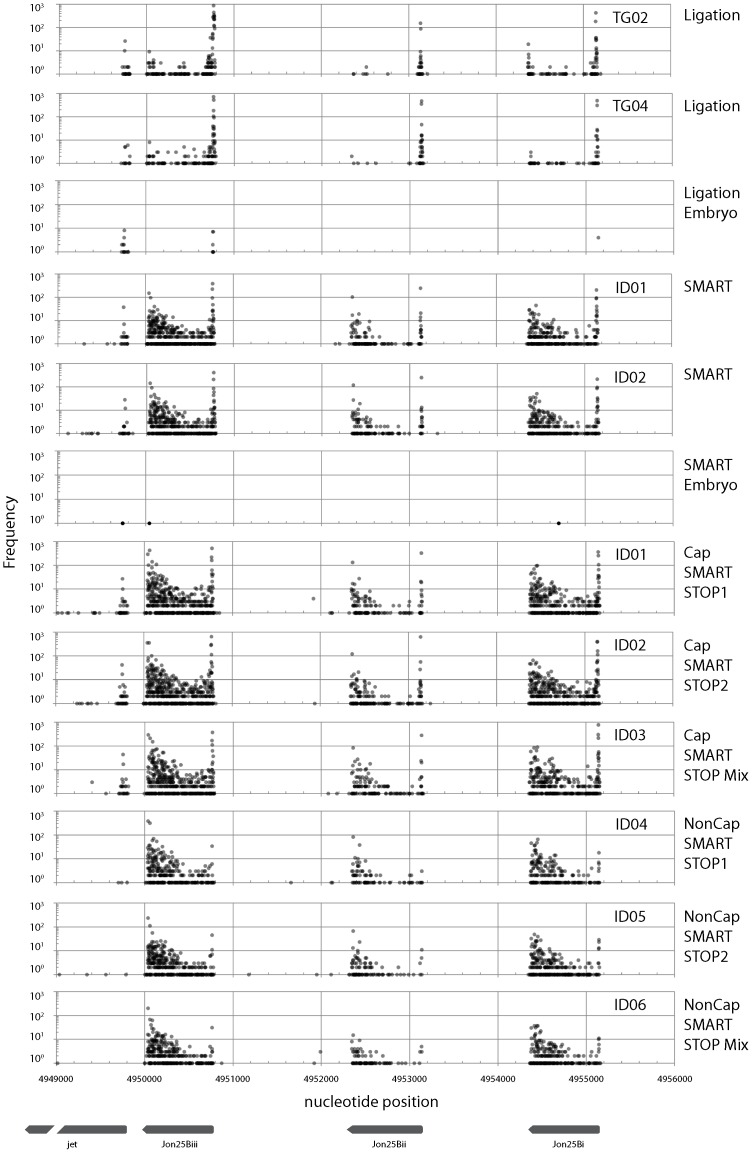
Frequency distribution of mapped sequence reads on the *Drosophila melanogaster* genome (Release 5) between nucleotide positions 4949000 and 4956000 on chromosome 2L. Only the sequences mapped on the minus strand are depicted. Gene locations (*Jon25Bi*, *Jon25Bii*, *Jon25Biii*, and *jet*) are depicted at the bottom of the figure. Plots from three libraries using SMART and ligation methods {adult (TG02, TG04, ID01, and ID02) and embryo RNA} and from three libraries using CapSMART and Non-CapSMART methods (ID01, ID02, ID03, ID04, ID05, and ID06) are depicted in the figure.

**Figure 9:**
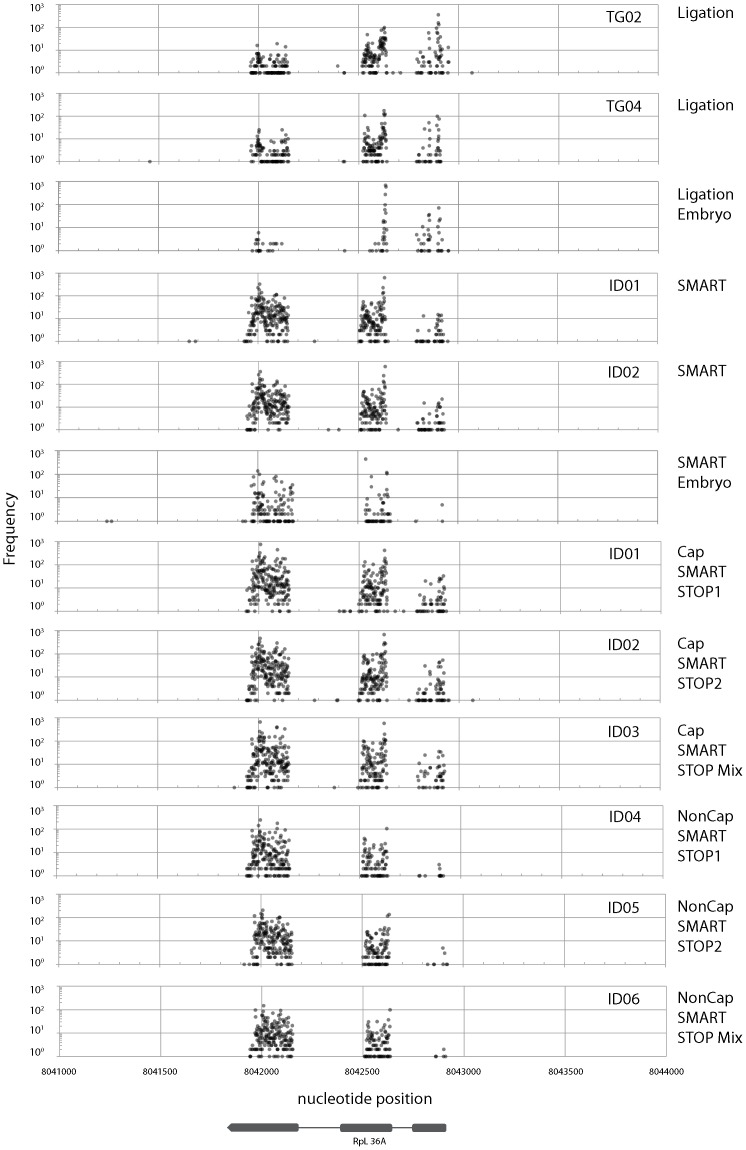
Frequency distribution of mapped sequence reads on the *Drosophila melanogaster* genome (Release 5) between nucleotide positions 8041000 and 8044000 on chromosome 2L. Only the sequences mapped on the minus strand are depicted. Gene location (*RpL36A*) is depicted at the bottom of the figure. Plots from three libraries using SMART and ligation methods {adult (TG02, TG04, ID01, and ID02) and embryo RNA} and from three libraries using CapSMART and Non-CapSMART methods (ID01, ID02, ID03, ID04, ID05, and ID06) are depicted in the figure.

Fragments obtained using all four methods were mapped onto the *Drosophila melanogaster* genome sequence (Release 5: [12]). Sequences mapped onto the minus strand of chromosome 2L were used for the following analyses. Three examples of the frequency distributions of mapped sequences are presented here (Figs. 7–9).

The first example (Fig. 7) encompasses the region surrounding *Rapgap1* and *CG13791* (FlyBase: [17]). In this region, large differences in frequency distribution were observed between libraries generated by different methods. No peaks were observed in libraries prepared by the ligation method. In contrast, two clear peaks were observed in the SMART library, which correspond to *Rapgap1* and *CG13791*. The second example (Fig. 8) is the region surrounding the *jet*, *Jon25Bi*, *Jon25Bii*, and *Jon25Biii* genes. Libraries constructed using adult poly A+ RNA as template revealed that this region contains three repeats with a right-facing “swan-shaped” distribution. Although the shape of the swans were similar in libraries derived from all four methods, the swan's bodies were smaller (i.e., the frequency distribution was reduced) in libraries prepared by the ligation method. Although we present only a few examples here, we observed several swan-shaped distributions in our dataset. In contrast to the libraries generated using adult poly A+ RNA, very low transcript frequencies were observed in libraries prepared using embryo poly A+ RNA. This observation is consistent with a previous report that gene transcription is lower at embryonic stages than in the adult [18]. The third example is the region surrounding the *RpL36A* gene (Fig. 9). In contrast to the other examples, the frequency distributions in this region were very similar when adult poly A+ RNA was used as template, regardless of library preparation method. On the contrary, large differences in frequency were observed between libraries prepared by different methods using embryonic poly A+ RNA as template (frequency was reduced at the left side peak for ligation-derived libraries, and at the right side peak in SMART method-derived libraries). In addition, the shape of the distribution was affected by the use of embryonic poly A+ RNA, as evidenced by the sharp central peak of libraries derived from the ligation, but not SMART method (Fig. 9).

The library preparation methods used in this study differ in that the SMART method does not require the 5′ cap structure (Fig. 1), while the ligation method does require it (Fig. 2). Therefore, peaks observed only in libraries generated by the SMART method represent 5′ ends without a cap structure. As such, these results indicate that a large proportion of mRNAs lack a 5′ cap structure. Until recently, decapping was believed to be an irreversible process that committed an mRNA molecule to degradation [19]. However, recent studies have indicated that recapping of mRNA may occur in the cytoplasm [20], [21]. Therefore, mRNAs without a cap structure may serve as a potential source of mRNA under certain conditions.

It is interesting to note that genes differed in the shape of their peaks. Swan-shaped distributions were observed for *Jon25Bi*, *Jon25Bii*, and *Jon25Biii* (Fig. 8). In contrast, sharp peaks were observed for the distributions of *CG13791* (Fig. 7) and *jet* (Fig. 8). Shapes also differed between developmental stages; for example, the central peak of *RpL36A* was considerably narrower in ligation libraries derived from embryonic RNA than those derived from adult RNA (Fig. 9). The 5′ untranslated regions play an important role in gene translation [22], [23], [24], but the underlying regulatory mechanisms are still largely unknown. Investigation of these mechanisms is beyond the scope of the present study. However, the methods described here will provide the means to elucidate such mechanisms.

Although lower reproducibility was observed using the ligation method (Fig. 6), mapping analyses revealed highly similar frequency distribution patterns between the libraries, irrespective of the tags used (Figs. 8 and 9: TG02 and TG04). This may be due to the wide range of transcription start sites, which normalize sequence-specific ligation bias. However, quantitative skews were sometimes observed at the transcription start sites of genes with a sharp peak distribution (such as *CG13791*, Fig. 7 and *jet*, Fig. 8). Therefore, we recommend the use of the same or a random tag sequence to facilitate comparisons, and the application of an Illumina-style “indexing” system for multiplexing [10]. An advantage of the ligation method is its high dependency on cap structure (see below). This high dependency enables us to determine the exact position of the transcription start site of mature capped mRNAs, which is not possible using the other three methods.

### Frequency of mitochondrial transcripts

The numbers of mitochondrial transcripts (which have no cap structure) obtained using the four library preparation methods are summarized in Tables S1, S2, and S3. We observed that few mitochondrial transcripts were sequenced using the ligation method, confirming that this method is highly dependent on cap structure.

Although very few mitochondrial transcripts were sequenced using the ligation method (Table S2), certain transcripts appeared to predominate over others. While the frequencies of *NADH dehydrogenase subunit* (*ND1*, *2*, *3*, *4*, *5*, and *6*) and *16S rRNA* transcripts were very low, higher frequencies were observed for transcripts of *Cytochrome oxidase subunit I*, *II*, and *III* (*CO1*, *CO2*, and *CO3*), *Cytochrome b apoenzyme* (*CYB*), and *ATP synthase subunit 8* (*ATP8*).

By modifying the SMART method, we developed two additional library preparation methods, CapSMART and Non-CapSMART. Both of these methods are based on ligation of non-natural nucleotides [25] to non-capped mRNA (CapSMART) or capped mRNA (Non-CapSMART) to suppress synthesis of non-target mRNA molecules (Fig. 3, 4). Although the frequency of mitochondrial transcripts indicated successful enrichment of target mRNA (fewer transcripts from CapSMART than from Non-CapSMART; Table S3), this pattern was not entirely consistent (e.g., a higher number of mitochondrial transcripts were observed in CapSMART ID03; Table S3). We hypothesize that this inconsistency may arise from ligation efficiency bias of non-natural nucleotides (iGiCiG and iCiGiC) to non-target molecules.

## Conclusions

We have developed four methods of using the Illumina platform to sequence mRNA 5′ ends. All four methods require small amounts of starting poly A+ RNA (minimum of 25 ng for the SMART method), and the entire library construction procedure can be completed in two to four days. Furthermore, all libraries were developed using commercially-available kits supplemented with additional oligos, making it easy for any laboratory to repeat these procedures. The SMART method outperformed the ligation method in terms of reproducibility, and therefore, this method is suitable for the quantification of mRNA abundance. In contrast, the ligation method is able to selectively sequence mRNAs with a 5′ cap structure. This latter technique promises to increase our understanding of the distribution of the 5′ end of genes. Finally, the resulting 5′ end profiles provide fresh insights into 5′ untranslated regions, indicating that mRNAs without a cap structure are abundant.

## Supporting Information

Table S1 Counts of mitochondrial transcripts obtained from libraries prepared by the SMART method (ID01–ID06) using adult poly A+ RNA. Mitochondrial 16S rRNA genes are known to be adenylated (Neira-Oviedo et al. 2011), and therefore their occurrence had been included for comparison purposes.
(XLSX)

Table S2 Counts of mitochondrial transcripts obtained from libraries prepared by the ligation method (TG02–TG12) using adult poly A+ RNA. Because of the low occurrence rates, parts-per notation were depicted as ppm in this table.
(XLSX)

Table S3 Mitochondrial transcripts obtained from libraries prepared by the CapSMART (ID01–ID03) and Non-CapSMART (ID04–ID06) methods using adult poly A+ RNA.
(XLSX)

## References

[1] Mortazavi A, Williams BA, McCue K, Schaeffer L, Wold B (2008) Mapping and quantifying mammalian transcriptomes by RNA-Seq. Nature Methods 5: 621–628. 18516045 10.1038/nmeth.1226 PMC13303166

[2] Nagalakshmi U, Wang Z, Waern K, Shou C, Raha D, et al (2008) The transcriptional landscape of the yeast genome defined by RNA sequencing. Science 320: 1344–1349. 18451266 10.1126/science.1158441 PMC2951732

[3] Shiroguchi K, Jia TZ, Sims PA, Xie XS (2012) Digital RNA sequencing minimizes sequence-dependent bias and amplification noise with optimized single-molecule barcodes. Proc Natl Acad Sci 109: 1347–1352. 22232676 10.1073/pnas.1118018109 PMC3268301

[4] Salimullah M, Sakai M, Plessy C, Carninci P (2011) NanoCAGE: A high-resolution technique to discover and interrogate cell transcriptomes. Cold Spring Harb Protoc doi:10.1101/pdb.prot5559 10.1101/pdb.prot5559 PMC4181851 21205859

[5] Takahashi H, Lassmann T, Murata M, Carninci P (2012) 5′ end-centered expression profiling using cap-analysis gene expression and next-generation sequencing. Nature Methods 7: 542–561. 10.1038/nprot.2012.005 PMC4094379 22362160

[6] Batut P, Dobin A, Plessy C, Carninci P (2013) High-fidelity promoter profiling reveals widespread alternative promoter usage and transposon-driven developmental gene expression. Genome Res 23: 169–180. 22936248 10.1101/gr.139618.112 PMC3530677

[7] Illumina, Inc. (2013) Using a PhiX control for HiSeq sequencing runs. Illumina Technical Note Sequencing.

[8] Zhu YY, Machleder EM, Chenchik A, Li R, Siebert PD (2001) Reverse transcriptase template switching: A SMART approach for full-length cDNA library construction. BioTechniques 30: 892–897. 11314272 10.2144/01304pf02

[9] Maruyama K, Sugano S (1994) Oligo-capping: a simple method to replace the cap structure of eukaryotic mRNAs with oligoribonucleotides. Gene 138: 171–174. 8125298 10.1016/0378-1119(94)90802-8

[10] Meyer M, Kircher M (2010) Illumina sequencing library preparation for highly multiplexed target capture and sequencing. Cold Spring Harbor Protoc doi:10.1101/pdb.prot5448 10.1101/pdb.prot5448 20516186

[11] Langmead B, Salzberg S (2012) Fast gapped-read alignment with Bowtie 2. Nature Methods 9: 357–359. 22388286 10.1038/nmeth.1923 PMC3322381

[12] Smith CD, Shu SQ, Mungall CJ, Karpen GH (2007) The release 5.1 annotation of *Drosophila melanogaster* heterochromatin. Science 316: 1586–91. 17569856 10.1126/science.1139815 PMC2819280

[13] Camacho C, Coulouris G, Avagyan V, Ma N, Papadopoulos J, et al (2009) BLAST+: architecture and applications. BMC Bioinformatics 10: 421. 20003500 10.1186/1471-2105-10-421 PMC2803857

[14] Alon S, Vigneault F, Eminaga S, Christodoulou DC, Seidman JG, et al (2011) Barcoding bias in high-throughput multiplex sequencing of miRNA. Genome Res 21: 1506–1511. 21750102 10.1101/gr.121715.111 PMC3166835

[15] Van Nieuwerburgh F, Soetaert S, Podshivalova K, Wang EAL, Schaffer L, et al (2011) Quantitative bias in Illumina TrueSeq and a novel post amplification barcoding strategy for multiplexed DNA and small RNA deep sequencing. PlosONE 6: e26969. 10.1371/journal.pone.0026969 PMC3203936 22046424

[16] Zhuang F, Fuchs RT, Sun Z, Zheng Y, Robb GB (2012) Structural bias in T4 RNA ligase-mediated 3′-adapter ligation. Nucl Acid Res 40: e54. 10.1093/nar/gkr1263 PMC3326334 22241775

[17] Marygold SJ, Leyland PC, Seal RL, Goodman JL, Thurmond JR, et al (2013) FlyBase: Improvement to the bibliography. Nucl Acid Res 41: D751–757. 10.1093/nar/gks1024 PMC3531214 23125371

[18] Carlson JR, Hogness DS (1985) Development and functional analysis of *Jonah* gene expression. Develop Biol 108: 355–368. 2416611 10.1016/0012-1606(85)90039-9

[19] Li Y, Kiledjian M (2010) Regulation of mRNA decapping. RNA 1: 253–265. 21935889 10.1002/wrna.15 PMC13110874

[20] Otsuka Y, Kedersha NL, Schoenberg DR (2009) Identification of a cytoplasmic complex that adds a cap onto 5′ –monophosphate RNA. Mol Cell Biol 29: 2155–2167. 19223470 10.1128/MCB.01325-08 PMC2663312

[21] Schoenberg DR, Maquat LE (2009) Re-capping the message. Trends Biochem Sci 34: 435–442. 19729311 10.1016/j.tibs.2009.05.003 PMC2743798

[22] Resch AM, Ogurtsov AY, Rogozin IB, Shabalina SA, Koonin EV (2009) Evolution of alternative and constitutive regions of mammalian 5′ UTRs. BMC Genomics 10: 162. 19371439 10.1186/1471-2164-10-162 PMC2674463

[23] Barrett LW, Fletcher S, Wilton SD (2012) Regulation of eukaryotic gene expression by the untranslated gene regions and other non-coding elements. Cell Mol Life Sci 69: 3613–3634. 22538991 10.1007/s00018-012-0990-9 PMC3474909

[24] Rojas-Duran MF, Gilbert WV (2013) Alternative transcription start site selection leads to large differences in translation activity in yeast. RNA 18: 2299–2305. 10.1261/rna.035865.112 PMC3504680 23105001

[25] Kapteyn J, He R, McDowell ET, Gang DR (2010) Incorporation of non-natural nucleotides into template-switching oligonucleotides reduces background and improves cDNA synthesis from very small RNA samples. BMC Genomics 11: 413. 20598146 10.1186/1471-2164-11-413 PMC2996941

